# Oral administration of *Lactobacillus paracasei* L9 attenuates PM_2.5_-induced enhancement of airway hyperresponsiveness and allergic airway response in murine model of asthma

**DOI:** 10.1371/journal.pone.0171721

**Published:** 2017-02-15

**Authors:** Xifan Wang, Yan Hui, Liang Zhao, Yanling Hao, Huiyuan Guo, Fazheng Ren

**Affiliations:** 1 Beijing Advanced Innovation Center for Food Nutrition and Human Health, China Agricultural University, Beijing, China; 2 Key Laboratory of Functional Dairy, Co-constructed by ministry of Education and Beijing Government, College of Food Science & Nutritional Engineering, China Agricultural University, Beijing, China; 3 Beijing Higher Institution Engineering Research Center of Animal Product, College of Food Science & Nutritional Engineering, China Agricultural University, Beijing, China; Centre National de la Recherche Scientifique, FRANCE

## Abstract

This study investigated allergy immunotherapy potential of *Lactobacillus paracasei* L9 to prevent or mitigate the particulate matter 2.5 (PM_2.5_) enhanced pre-existing asthma in mice. Firstly, we used a mouse model of asthma (a 21-day ovalbumin (OVA) sensitization and challenge model) followed by PM_2.5_ exposure twice on the same day of the last challenge. PM_2.5_ was collected from the urban area of Beijing and underwent analysis for metals and polycyclic aromatic hydrocarbon contents. The results showed that PM_2.5_ exposure enhanced airway hyper-responsiveness (AHR) and lead to a mixed Th2/ IL-17 response in asthmatic mice. Secondly, the PM_2.5_ exposed asthmatic mice were orally administered with L9 (4×10^7^, 4×10^9^ CFU/mouse, day) from the day of first sensitization to the endpoint, for 20 days, to investigate the potential mitigative effect of L9 on asthma. The results showed that L9 ameliorated PM_2.5_ exposure enhanced AHR with an approximate 50% decrease in total airway resistance response to methacholine (48 mg/ml). L9 also prevented the exacerbated eosinophil and neutrophil infiltration in bronchoalveolar lavage fluid (BALF), and decreased the serum level of total IgE and OVA-specific IgG1 by 0.44-fold and 0.3-fold, respectively. Additionally, cytokine production showed that L9 significantly decreased T-helper cell type 2 (Th2)–related cytokines (IL-4, -5, -13) and elevated levels of Th1 related IFN-γ in BALF. L9 also reduced the level of IL-17A and increased the level of TGF-β. Taken together, these results indicate that L9 may exert the anti-allergic benefit, possibly through rebalancing Th1/Th2 immune response and modulating IL-17 pro-inflammatory immune response. Thus, L9 is a promising candidate for preventing PM exposure enhanced pre-existing asthma.

## Introduction

In recent years, China has experienced frequent and extremely severe smog, especially in larger cities, like Beijing. Particulate matter (PM) with an aerodynamic diameter less than 2.5 μm (PM_2.5_) is the major pollutant causing this haze pollution [1]. The highest daily average PM_2.5_ concentration in Beijing measured is greater than 500 μg/m^3^ at times, which was 20-fold higher than the World Health Organization (WHO) recommended value [2]. In conjunction with this increase in air particulate matter, the incidence of wide-spread respiratory irritation symptoms and hospital visits have also significantly increased. Compared with winter reference periods (December 27–30, 2012, and January 21–24, 2013), a statistically significant increase (risk ratios: 1.16) in outpatient medical visits for respiratory diseases, like asthma, was observed during the heavy smog period of January 10–17, 2013 [3].

Asthma is a heterogeneous disease characterized by varying levels of reversible airflow obstruction, airway hyper-responsiveness (AHR), mucus secretion and chronic inflammation [4, 5]. Several studies have demonstrated that a combination of genetic, epigenetic and environmental factors contribute to asthma heterogeneity [4, 6, 7]. Indeed, large-scale epidemiologic and experimental studies have shown that exposure to outdoor air pollution, such as particulate matter (PM), increases the risk of exacerbations of pre-existing asthma [8–10]. The classic asthma presentation is generally regarded as a T helper cell type 2 (Th2) airway inflammation, as high levels of eosinophil, total immunoglobulin (Ig) E and Th2 cell-related interleukin (IL)-4, -5, -13 were observed. However, particulate matter has been shown to induce new cellular and molecular mediators such as Th17 cells and IL-17A in the lungs of exposed mice in recent studies [11, 12]. T-helper 17 (Th17) cells, a CD4^+^ helper T cell subset that produces interleukin-17a (IL-17A) have been discovered to play important roles in more severe asthma phenotypes. Importantly, asthmatics with an overexpression of IL-17A and neutrophilia proved to have the lowest lung function and the worst asthma control when compared to other subsets [13, 14]. Thus, it is critical to find effective preventive strategies for those diagnosed with pre-existing asthma who are also consistently exposed to serious air pollution.

Probiotics such as lactobacilli and bifidobacteria have been reported to alleviate asthmatic symptoms [15–17]. These probiotics provide benefits by promoting T regulatory (Treg) cell development and rebalancing Th1/Th2 responses toward a Th1-dominant state [16, 18]. For example, live *Lactobacillus paracasei* KW3110 administered orally to ovalbumin (OVA) -allergic mice revealed anti-allergic effects on both Th1 and Th2 cytokines, IL-12 induction and IL-4 repression [19]. Oral administration of *Lactobacillus rhamnosus* (Lcr35) was reported to attenuate the features of allergic asthma in a mouse model, and induce immune regulation by a CD4^+^CD25^+^Foxp3^+^Treg cell-mediated process. [20]. Moreover, a recent study demonstrated that *Lactobacillus gasseri* can suppress Th17 pro-inflammatory response and inhibit OVA-induced airway inflammation in mice [21]. However, the previous studies only assessed the beneficial effects of probiotics on a classical allergen-induced mouse model of asthma, typically induced by OVA or house dust mite. Whether or not probiotics will have benefits for mixed and severe asthmatic responses induced by PM_2.5_ exposure still remains unclear.

*Lactobacillus paracasei* L9 (L9) originally isolated from the feces of healthy centenarians has been demonstrated to attenuate the symptoms of food allergy in a murine model by inducing Treg cells associated with increased TGF-β production [22]. In this study, we evaluate the allergy immunotherapy potential of L9 to prevent PM_2.5_ exposure enhanced AHR and allergic response in asthmatic mice. We first collected the ambient PM_2.5_ in Beijing and set up a murine model to investigate the effect of PM_2.5_ exposure on pre-existing asthma induced by OVA. Metal and PAH components of the ambient PM_2.5_ were tested. We then assessed the capability of L9 to modulate the PM_2.5_ exposure enhanced allergic response, and the mechanism by which L9 modulated the immune system.

## Materials and methods

### Preparation of *Lactobacillus paracasei* L9

L9 was grown in MRS broth anaerobically at 37°C. The bacteria were incubated overnight and harvested by centrifuge at 3000 × g for 15 min at 4°C, before being resuspended in sterile phosphate buffer saline (PBS). Bacterial concentration and viability were determined by plate counts.

### Preparation of PM_2.5_

Airborne particular matter, less than 2.5 μm in aerodynamic diameter, was collected with fiber glass filters (Φ80 mm, Laoying, Qingdao, China) [23] by means of a high-volume air sampler (Laoying 2050, Qingdao, China) from November 26, 2014 to February 21, 2015 in Beijing. All filters were equilibrated in a condition of 30% relative humidity in 25°C room temperature for over 48 h and then weighed on a high-precision microbalance to measure a daily atmospheric PM_2.5_ concentration. PM_2.5_ was gathered at a flow rate of 100 L/min for a continuous 8 hour period. The collected fiber filters were cut into pieces and immersed in Millipore water. Particles were extracted from the water by sonication using ultrasound frequencies for 1 hour on ice. The water with extracted PM_2.5_ was centrifuged at 5000 × g, 4°C, and then concentrated by freeze-drying. The particles were stored at -20°C after adding 0.85% NaCl to a final concentration of 12 μg/μl. The extracts of unused filters were prepared using the same method as a control.

### Measurement of components of PM_2.5_

Metals of the ambient PM_2.5_ dissolved in nitric acid were analyzed by Inductively Coupled Plasma-Atomic Emission Spectroscopy (ICP-AES, Thermo ICAP 6300, Thermo Fisher Scientific Inc., USA). Several metals with relatively lower concentration (Cd, Cr, Cu, Ni, Pb, Se, V) were analyzed again by Inductively Coupled Plasma-Mass Spectrometry (ICP-MS, Agilent 7900, Agilent Technologies, Palo Alto, CA, USA) to ensure accuracy.

Polynuclear aromatic hydrocarbons (PAHs) were eluted from PM_2.5_ extraction and control filter extraction using ether and n-hexane (v: v = 1:1) in a Soxhlet extractor. The presence of PAHs was determined by using a 6890 gas chromatograph equipped with a 5977 mass spectrometer (GC-MS, Agilent Technologies, Palo Alto, CA, USA) according to Chinese standard procedure (HJ 646–2013).

### Establishment of a PM_2.5_ enhanced mouse model of asthma and probiotics treatment

Animal experiments were approved by the Animal Care and Use Committee of China Agriculture University. Six-week-old female BALB/c mice were purchased from Charles River Breeding Laboratories (Beijing, China), and maintained in a temperature and humidity-controlled specific-pathogen-free (SPF) room (at 25 ± 2°C) on a 12 hour light, 12 hour dark (12L:12D) schedule. Mice were fed a standard mouse chow containing no OVA or microbes (Charles River Breeding Laboratories). Mice were checked every day and the activity situation and body weight were observed for animal health monitoring. There was no animal became severely ill or died before the experimental endpoint.

**Fig 1:**
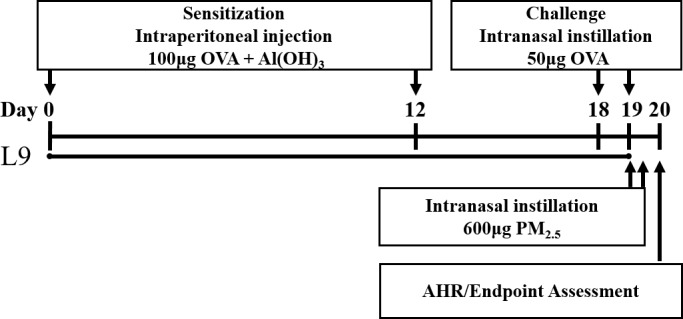
Experimental setup of the PM_2.5_ exposure enhanced mouse model of asthma.

The mouse model was based on a classic 21-day OVA-sensitized and challenged mouse model of asthma [24], followed by the intranasal instillation of the PM_2.5_ extracted solution (Fig 1). After 4 days acclimatization, mice were assigned to one of four groups (n = 8/group): the PBS-sensitized and challenged control group (CON), the OVA-sensitized and challenged group (OVA), the control filter extract exposed OVA group (OVA +Filter), and the PM_2.5_ extract exposed OVA group (OVA +PM_2.5_). All groups except for the CON group were intraperitoneally injected with 200 μL of aluminum hydroxide (Al(OH)_3_) and saline (1:1) containing 100 μg OVA (Sigma-Aldrich, Beijing, China) on day 0 and 12, while the CON received Al(OH)_3_ and saline as a control. On day 18 and 19, the CON group and the other groups were challenged with either 50μL saline or the same dose of OVA (50μg/50μL per mouse) by intranasal instillation. For OVA+PM_2.5_ group, after the last OVA challenge on day 19, mice were given an extra intranasal administration of 50μL PM_2.5_ solution (600μg/50μL per mouse), twice, over an interval of 4 hours. At the same time, the same dose of control filter extracted solution was intranasally administered to the OVA+FILTER group. All mice were under anesthesia with isoflurane when given intranasal instillation.

Based on the PM_2.5_ enhanced OVA-induced mouse model of asthma, mice were fed either L9 or the same volume of PBS by gavage from the day of first sensitization (day 0) to the endpoint, for 20 days during sensitization (Fig 1). The L9 fed mice (L9-L/P, L9-H/P) were gavaged with lower or higher doses of L9 in 200μL PBS (4×10^7^, 4×10^9^ CFU/mouse per day, respectively), using an aseptic gavage tube. Meanwhile, the other four groups (CON, OVA, OVA+FILTER and OVA+PM_2.5_) were gavaged with PBS (200 μL/mouse per day) as a placebo.

### Measurement of airway hyper-responsiveness

Measurements of AHR were taken twenty-four hours after the last challenge on day 20 by the FlexiVent system (Scireq Inc., Montreal, Canada) as previously described [24]. In brief, mice were tracheostomized and ventilated under anesthetization with avertin (500 mg/kg body weight), then intubated with an18-gauge stainless steel cannula. The pulmonary mechanics were measured at a rate of 150 breaths/min with a tidal volume of 8 mL/kg and positive end expiratory pressure of 2.0 cm H_2_O, as set on the FlexiVent system. Mice were challenged by normal saline (for the baseline measurement) and methacholine (Mch) (0.75, 1.5, 3, 6, 12, 24, 48 mg/mL) via a matched nebulizer. After each dose, the response was measured by applying 2-s perturbations at 10-s intervals for a total of 3 min. The dose-response curves for each groups were determined and the total respiratory system resistance (Rrs) were described below.

### Analysis of cell composition of bronchoalveolar lavage fluid

Bronchoalveolar lavage fluid (BALF) was collected from mice immediately following euthanasia by cervical dislocation.as previously described [24]. The BALF was placed on ice and centrifuged at 1500 rpm for 10 minutes at 4°C. The supernatants were collected for cytokine analysis and the sediments suspended with PBS were used for a cell composition assessment. Cell counts of macrophages, eosinophils, neutrophils and lymphocytes were performed by counting at least 200 cells in the suspended BALF stained with hematoxylin (Beijing ZSGB-BIO Technology Co Ltd, Beijing, China) and Congo red (Sigma-Aldrich, Beijing, China) staining.

### ELISA for BALF cytokines and serum immunoglobulins

The concentrations of IL-4, IL-5, IL-13, IFN-γ, IL-17A and TGF-β in BALF were analyzed with commercial ELISA kits (eBioscience, Boston, MA, USA) according to the manufacturers’ instructions. Blood was collected using retro-venous plexus punctures and serum was separated by centrifugation (5000 rpm, 4°C, 20 min) after resting at 4°C overnight. The levels of total IgE, IgG1 and IgG2a in serum were measured using the same brand of commercial ELISA kits. OVA-specific IgG1 and IgG2 in serum were tested by ELISA with revised method based on published papers [25–27]. To be more specific, 96-well plates (Corning Costar, 9018) were coated with 20μg/mL OVA in coating buffer (eBioscience) overnight at 4°C and then incubated at room temperature for 2h with 1% BSA in PBS for blocking. After washing, diluted samples were added to a microplate and incubated at 4°C overnight. After another washing, goat anti-mouse IgG1 and IgG2a conjugated with horseradish peroxidase (abcam) was used for detection, respectively, and then placed the plate at room temperature for 2h. Tetramethybenzidine substrate solution (eBioscience) was added and incubated for 15min at room temperature and stopped by 4N H_2_SO_4_. The optical density was measured at 450 nm.

### Histological experiment of lung tissue

Left lung lobes of all subjects were immediately removed after lavage and fixed in 10% formalin for 24 h at room temperature and then embedded in paraffin. The embedded tissue was then stained with hematoxylin and eosin (H&E) to evaluate morphology and inflammation using a light microscope (Zeiss Inc., Germany).

### Statistical analysis

Data were expressed as mean presented as the mean ± SEM. The differences between experimental groups were analyzed using SPSS Statistics 20. Binary comparisons were made by using the student *t* test. Comparisons between multiple groups were assessed using a one-way ANOVA followed by an LSD-test. For all tests, *P*-values less than 0.05 were considered significantly.

## Results

### Analysis of the components of PM_2.5_

Metals and PAHs are the most suspicious components related to airway inflammation and physiologic responses in both animal and humans studies. A total of 17 metals in PM_2.5_ samples were measured by ICP-MAS or ICP-AES (Table 1). The results showed that Ca, K, Mg, Al, and Zn were the prevalent metals found in ambient PM_2.5_. Compared with the extract of control filter, the concentration of Pb, Mn, Cu and As in the extract of PM_2.5_ were 1343, 162, 155 and 59 times higher, respectively. Furthermore, BaP, BaA, BbFA, CHR, BPE, IPY and PYR were the main constituents of PAHs found in ambient PM_2.5_. None of these hydrocarbons were found in the filter samples. Thus, the high concentration of transition metals and PAHs in the ambient PM_2.5_ may account for the exacerbation effect of PM_2.5_ on pre-existing asthma.

**Table 1:** Analysis of Metal and PAH composition in PM_2.5_.

Metal	Control Filter (ug/g)	PM_2.5_ (ug/g)	PAH	Control Filter (ug/g)	PM_2.5_ (ug/g)
Al	1346.25	18166.7	Benzo[a]pyrene (BaP)	N.D.	9.95
As	5.42	321.7	Benzo[a]anthracene (BaA)	N.D.	12.35
Ba	272.92	5460.83	Dibenzo[a,h]anthracene (DBA)	N.D.	1.59
Ca	3665.83	70641.7	Benzo[b]fluoranthene (BbFA)	N.D.	18.65
Cd	N.D.	22.5	Benzo[k] fluoranthene (BkF)	N.D.	4.025
Cr	1.06	49.92	Chrysene (CHR)	N.D.	11
Cu	2.56	396.83	Acenaphthene (ANA)	N.D.	0.1505
Fe	86.71	558.58	Acenaphthylene (ANY)	N.D.	0.399
K	285.75	20508.3	Anthracene (ANT)	N.D.	0.221
Mg	977.08	18933.3	Benzo[ghi]perylene (BPE)	N.D.	7.95
Mn	3.13	505.08	Fluoranthene (FLT)	N.D.	4.445
Ni	1.17	20	Fluorene (FLU)	N.D.	0.191
Pb	0.42	559.5	Indeno[1,2,3-cd]pyrene (IPY)	N.D.	9.7
Se	3.38	50.33	Naphthalene (NAP)	N.D.	0.156
Ti	38.33	466.17	Phenanthrene (PHE)	N.D.	1.025
V	5.25	73.75	Pyrene (PYR)	N.D.	12.65
Zn	183.33	8018.33			

### PM_2.5_ exposure exacerbated AHR in asthmatic mice

**Fig 2:**
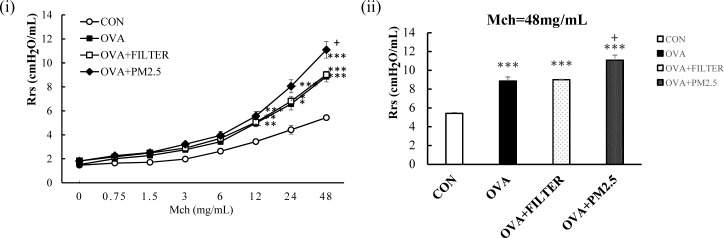
PM_2.5_ exposure exacerbated AHR in mice with OVA induced pre-existing asthma. (A) AHR to increasing doses of Mch. (B) The maximal response to Mch challenge. Each value is expressed as mean ± SEM. N = 5–8. * *P*< 0.05, ** *P* < 0.005, *** *P* < 0.001 vs. CON; ^+^ *P* < 0.05 vs. OVA+FILTER.

After the last OVA challenge, mice were intranasally exposed to control filter extract or PM_2.5_ extract twice before an AHR test. As shown in Fig 2, OVA mice exhibited moderately increased AHR in response to increasing doses of MCh when compared with the control mice. When the nebulized MCh reached the maximal dose, the Rrs of the OVA mice was a significant 1.63-fold higher than that of the CON mice (*P*<0.001). No changes were observed after the intranasal administration of control filter extract in OVA mice. PM_2.5_ exposed mice showed significantly enhanced Rrs (1.25-fold) when compared with OVA+Filter mice or OVA mice (*P*<0.05). Thus, PM_2.5_ exposure significantly exacerbated airway responsiveness induced by OVA.

### PM_2.5_ exposure induced inflammatory cell infiltration in lungs of asthmatic mice

**Fig 3:**
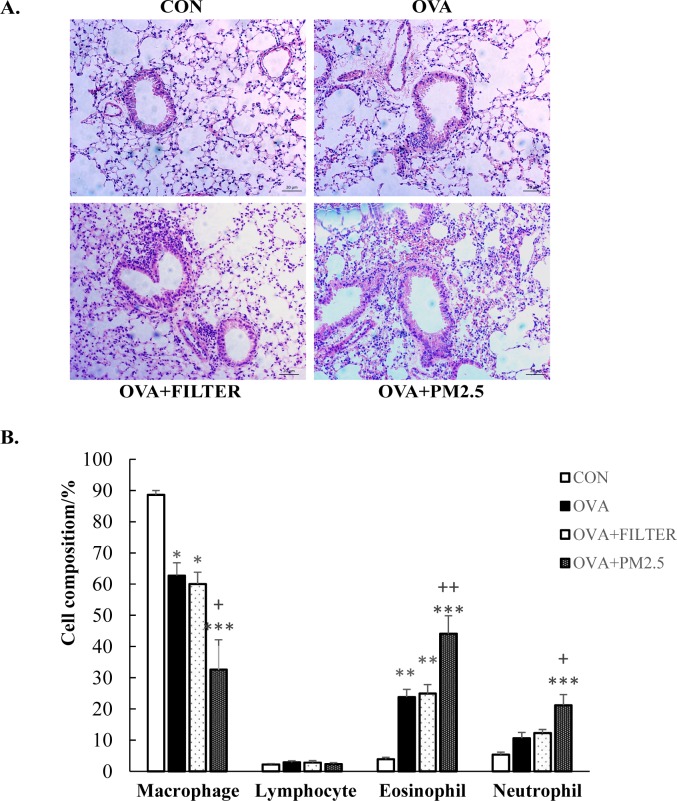
PM_2.5_ exposure exacerbated airway inflammatory cell infiltration in mice with OVA induced pre-existing asthma. (A) Histopathological examination of lung tissue inflammatory cell infiltration in mice. Representative photos of H&E-stained lung sections (original magnification 20x). (B) Cell population of macrophage, eosinophil, neutrophil and lymphocyte in BALF. Each value is expressed as mean ± SEM. n = 5–8. * *P*< 0.05, ** *P* < 0.005, *** *P* < 0.001 vs. CON; ^+^ *P* < 0.05 vs. OVA+FILTER.

The effect of PM_2.5_ exposure on the lungs of mice with established asthma was further evaluated by histological examination (Fig 3A). OVA sensitization and challenge caused the presence of inflammatory cell infiltrates. The cell infiltration in the peribronchial and perivascular regions was more severe in OVA+PM_2.5_ mice than that in the OVA mice. In order to further evaluate the lung inflammation in PM_2.5_ exposed asthmatic mice, the numbers of macrophages, eosinophils, neutrophils and lymphocytes were counted to obtain the cell composition of the BALF. As shown in Fig 3B, the percentage of eosinophils in OVA mice was significantly higher than that in the CON mice (*P*<0.005). There is, however, no significant difference between the CON mice and OVA mice in the number of present neutrophils. There was also no significant difference between the OVA mice and the OVA+FILTER mice. Compared to OVA mice, exposure to PM_2.5_ increased the number of both eosinophils and neutrophils approximately 2-fold (*P*<0.005). Thus, PM_2.5_ exposure exacerbated the airway inflammation in asthmatic mice.

### PM_2.5_ exposure exacerbated systemic and airway allergic response in asthmatic mice

**Fig 4:**
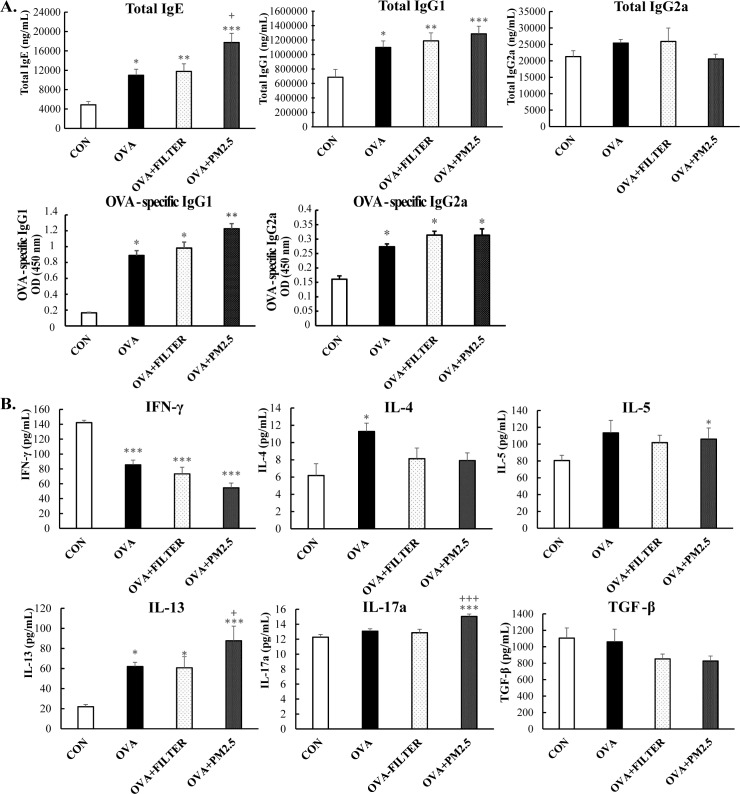
PM_2.5_ exposure exacerbated systemic and airway allergic response in mice with OVA induced pre-existing asthma. (A) Serum levels of immunoglobulin production. (B) Cytokines production in BALF. Each value is expressed as mean ± SEM. n = 5–8. * *P*< 0.05, ** *P* < 0.005, *** *P* < 0.001 vs. CON; ^+^ *P* < 0.05 vs. OVA+FILTER.

The levels of serum immunoglobulins were investigated to elucidate the effect of PM_2.5_ exposure on systemic allergic response. As shown in Fig 4A, the total serum IgE and IgG1, as well as the OVA-specific IgG1 and IgG2a in the OVA mice were significantly higher than those in the control mice (*P* <0.05). PM_2.5_ exposure increased the level of total serum IgE by 1.62 fold and moderately increased the level of total serum IgG1and OVA-specific IgG1, when compared to the OVA mice. There were no significant differences between the OVA mice and PM2.5 mice in the level of total IgG2a and OVA-specific IgG2a. The exposure to filter extraction did not lead to any significant difference when compared to OVA mice. In addition, the cytokines in BALF were also evaluated to clarify the effect of PM_2.5_ exposure on airway allergic response. As shown in Fig 4B, when compared to the control mice, the levels of Th2- related cytokines (IL-4 and IL-13) were significantly increased (*P*<0.05, *P*<0.05), while level of Th1-related cytokines (IFN-γ) were significantly decreased in the BALF of the OVA mice (*P*<0.001). The exposure of filter extraction, however, did not demonstrate any difference in OVA mice, though the exposure of PM_2.5_ significantly increased the concentrations of IL-13 by 1.41-fold (*P*<0.05) while inducing the production of IL-17A in the BALF (*P*<0.001). These results suggest that PM_2.5_ exposure may exacerbate pre-existing asthma by inducing a mixed Th2 and IL-17 response.

### Administration of *Lactobacillus paracasei* L9 attenuated the exacerbated AHR induced by PM_2.5_ exposure

**Fig 5:**
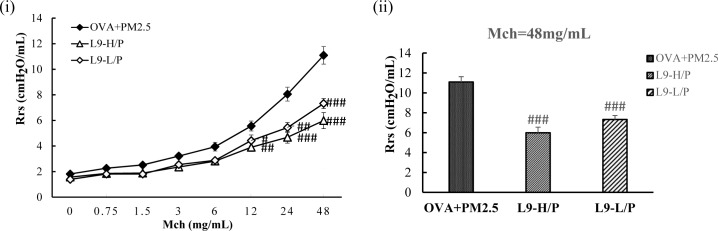
Administration of L9 ameliorated AHR enhanced by exposure of PM_2.5_. (A) AHR to increasing doses of Mch. (B) The maximal response to Mch challenge. Each value is expressed as mean ± SEM. n = 5–8. ## *P* < 0.005, ### *P* < 0.001 vs. OVA+PM_2.5_.

The effect of oral administration of L9 on PM_2.5_ exposure enhanced AHR was evaluated (Fig 5). The results showed that a daily oral administration of L9 (4×10^7^, 4×10^9^ CFU/mouse) significantly (*P* < 0.001) alleviated the enhanced Rrs in OVA+PM_2.5_ mice. Specifically, the maximal response to the MCh challenge (48mg/ml) was ameliorated with an approximate 50% decrease in L9-H/P (higher dose) mice. The maximal Rrs of L9-L/P (lower dose) mice was also decreased when compared to that of OVA+PM_2.5_ mice, but significantly higher than that of L9-H/P (higher dose) mice (*P* <0.05). Thus, administration of L9 attenuated the exacerbated AHR induced by PM_2.5_ exposure in asthmatic mice.

### Administration of *Lactobacillus paracasei* L9 prevented exacerbation of inflammatory cell infiltration

**Fig 6:**
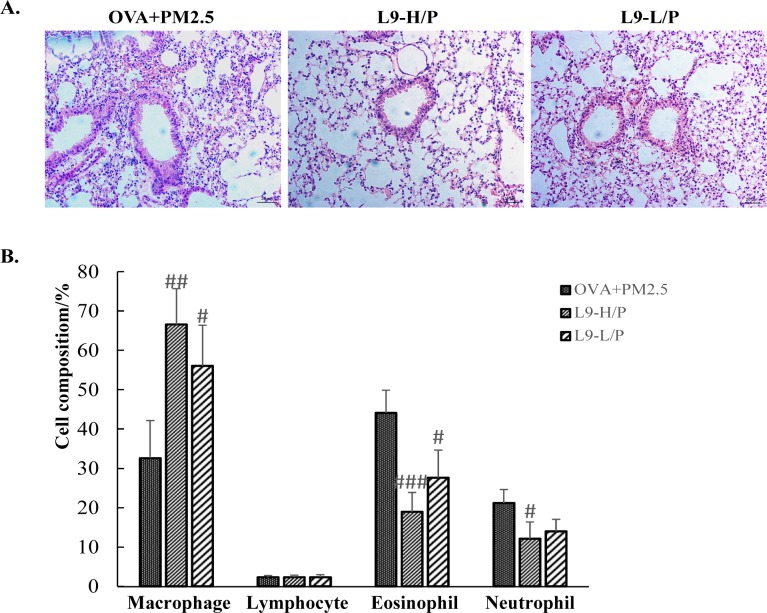
Administration of L9 prevented exacerbated inflammatory cell infiltration induced by exposure of PM_2.5_. (A) Histopathological examination of lung tissue inflammatory cell infiltration in mice. Representative photos of H&E-stained lung sections (original magnification 20x). (B) Cell population of macrophage, eosinophil, neutrophil and lymphocyte in BALF. Each value is expressed as mean ± SEM. n = 5–8. ## *P* < 0.005, ### *P* < 0.001 vs. OVA+PM_2.5_.

The exacerbation of inflammatory cell infiltration was prevented in mice with the oral administration of L9 on H&E staining, when compared with the OVA+PM_2.5_ mice (Fig 6A). Higher doses of L9 seemed to demonstrate better efficacy in alleviating cell infiltration in the peribronchial and perivascular regions than did lower doses. Furthermore, after the administration of a higher dose of L9, the percentage of eosinophils and neutrophils in BALF were significantly decreased by 0.43-fold and 0.57-fold, respectively, in comparison to those in the OVA+PM_2.5_ mice (*P* <0.001; *P* <0.05). A lower dose of L9 can also significantly decrease cell concentrations of eosinophils (*P* <0.05), but exhibited less of an ability to decrease cell concentrations of neutrophils (Fig 6B). Thus, administration of L9 can improve PM_2.5_ exposure induced inflammatory cell infiltration in the lungs of mice, but the effects are dependent on dose.

### Administration of *Lactobacillus paracasei* L9 attenuated the mixed allergic response induced by PM_2.5_ exposure

**Fig 7:**
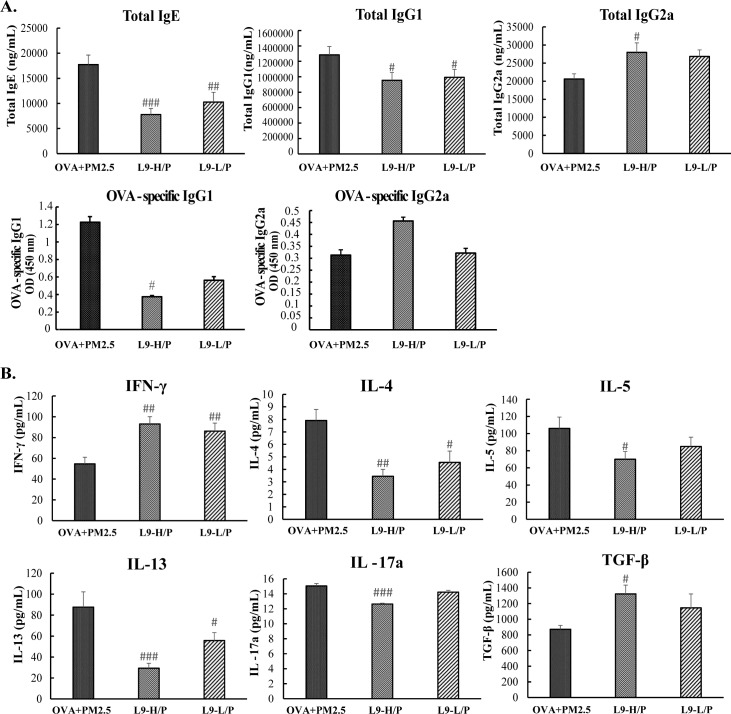
Administration of L9 attenuated the mixed allergic response induced by exposure of PM_2.5_. (A) Serum levels of immunoglobulin production. (B) Cytokines production in BALF. Each value is expressed as mean ± SEM. n = 5–8. # *P* < 0.05, ## *P* < 0.005, ### *P* < 0.001 vs. OVA+PM_2.5_.

The systemic allergic response was also attenuated after oral administration of L9. As shown in Fig 7A, the administration of a higher dose of L9 significantly decreased the concentration of serum total IgE and IgG1 (*P* <0.001, *P* <0.05), while significantly increasing the level of serum IgG2a. (*P* <0.05). Moreover, higher dose of L9 significantly decreased the level of OVA-specific IgG1 by 0.3 fold (*P* <0.05) while showing a tendency to increase the level of OVA-specific IgG2a. The effect of L9 on these immune globulins seems to be dose-dependent. Furthermore, the administration of a higher dose of L9 significantly decreased the level of Th2-related cytokines, IL-4, 5, 13 to nearly half (*P* <0.005, *P* <0.05, *P* <0.001), but significantly increased the level of Th1-related cytokines, IFN-γ with 1.71-fold (*P* <0.005) in BALF (Fig 7B). In addition, a higher dose of L9 also significantly mitigated the level of IL-17A induced by PM_2.5_ exposure (*P* <0.001) (Fig 7B). Moreover, the level of TGF-β, which is thought to be predominantly produced by Treg cells, was significantly increased in L9-H/P mice (Fig 7B). Thus, oral administration of L9 attenuated the mixed Th2 and IL-17 allergic airway response induced by exposure of PM_2.5_ in asthmatic mice. Higher dose of L9 showed more significant effects on attenuating the mixed inflammatory response than did lower dose.

## Discussion

The present study investigated the effect of orally administered probiotics, specifically *Lactobacillus paracasei* L9, to prevent or mitigate the exacerbated AHR and allergic response attributed to PM_2.5_ exposure in asthmatic mice. As we known, our study is the first time to explore the effect of oral intaking probiotics on PM2.5 exposure enhanced pre-existing asthma.

PM_2.5_ exposure induced an increased IL-17A level and the accumulation of neutrophils in the lungs of asthmatic mice, except for the exacerbation of Th2-related response. Recently, several lines of evidence suggest that IL-17A plays an important role during the pathophysiological process of allergic airway inflammation, especially in neutrophilic inflammation [28, 29]. Several cell types might contribute to production of IL-17A, including ILC3, CD8^+^ T cells, NK cells, and γδT cells [30–32]. But Th17, the third subset of Th cells, has been considered as the primary producer of IL-17A [33, 34]. These data suggest that individuals with pre-existing asthma are susceptible to developing more severe asthma with a mixed Th2 and Th17 response when exposed to ambient PM_2.5_. These findings are consistent with previous studies which reported that exposure to ambient PM or diesel exhaust particulate (DEP) induce IL-17A production and lead to mixed inflammatory asthmatic phenotype including both eosinophil and neutrophil infiltration in lung cells of exposed mice [11, 12, 35]. Th17 cell and IL-17A is proven to promote the secretion of neutrophil chemokines by epithelial cells, to exacerbate AHR by directly promoting airway smooth muscle contraction, and to enhance neutrophilic airway inflammation and Th2 cell-mediated eosinophilic airway inflammation in a murine asthma model [36–38]. Additionally, it has been recently demonstrated that IL-17A levels in bronchial biopsies from asthma patients are associated with disease severity [14]. Thus, the increased IL-17A attribution to PM_2.5_ exposure in this study plays a vital role in these severe asthma symptoms.

Metals and PAHs have been thought to be the most harmful components in PM_2.5_. The direct effects of metal composition of ambient air PM_2.5_ on subsequent allergic responses have been commonly reported [39–41]. In this study, the PM_2.5_ samples we collected in Beijing contain high concentrations of metal elements, especially of As, Cu, Cr, Mn, Pb, Zn, K. Several of these metals have been demonstrated to affect airway inflammation and physiologic responses in both animal and epidemiologic studies [39, 42, 43]. Gavett et al. reported that higher concentrations of many transition and toxic metals, including zinc, lead, copper, cadmium, tin, and arsenic were associated with increasing Th2-related cytokines [44]. Accordingly, the contribution of metals in PM-induced pulmonary injury was also proven to be associated with an increase in eosinophils [45]. Thus, high concentrations of transition and toxic metal in the ambient PM_2.5_ may account for the increased Th2 response after exposure to PM_2.5_. Furthermore, the Th17‑cell activation might be driven by PAHs in the PM_2.5_. In this study, BaP, BaA, BbFA, CHR, BPE, IPY, PYR were extremely high in the PM samples. Recently, several studies have described a role of the aryl hydrocarbon receptor, a primary receptor for PAHs in T cells, in the regulation of Th17 differentiation [46, 47]. Knopp et al. proved a novel mechanism in which PAHs contained in PM can directly act on the aryl hydrocarbon receptor in T cells, leading to enhanced Th17 differentiation [48]. Thus, it can be inferred from this finding that the increase of IL-17A after PM_2.5_ exposure in the present study can be attributed to high concentrations of PAHs in the ambient PM_2.5_.

The protective effect of probiotics (or LAB) in inflammatory disorders has gained worldwide attention. There are increasing clinical evidences of LAB to prevent or treat inflammatory bowel disease [49] and allergic disease [50–52]. As for their anti-allergy mechanisms, different species or strains of LAB may exhibit different responses. Heat-killed *L*. *plantarum* KTCT 3104 was reported to reduce OVA-induced AHR by the reduction of Th2-related cytokines, IL-4 and IL-5, and an enhancement of Th1-related cytokine, IFN-γ [53]. However, Oral application *Lactobacillus rhamnosus* GG inhibited allergic sensitization and airway disease in a murine model of asthma by induction of Treg cells, associated with a parallel suppression of the classical Th1-related cytokine (IFN-γ) and Th2-related cytokines (IL-4 and IL-5) [54]. Hougee et al. compared the effects of *Bifidobacterium breve* M-16V and *Lactobacillus plantarum* NumRes8 in OVA-sensitized mice. Results showed that both bacteria reduced the numbers of eosinophils and lowered the levels of OVA-specific IgE, but only B. breve M-16V can reduce the secretion of Th2-related cytokines (IL-4 and IL-5)[55].

According to our data, the oral administration of L9 shows a great ability to moderate AHR, airway inflammation and a mixed Th2 and IL-17 response induced by PM_2.5_ and OVA co-exposure. The cytokines produced in BALF show that L9 dose-dependently decreases the Th2-related cytokines (IL-4, 5, 13) level, but increases the Th1-related cytokines (IFN-γ). These were similar to results observed in studies of probiotics to modulate ovalbumin (OVA) induced asthma. Liu et al. investigated the anti-allergic effects of *Lactobacillus plantarum* K37 (K37) on airway hyper-responsiveness (AHR) and systemic allergic responses, and found that K37 effectively alleviated the allergic responses in OVA-sensitized and challenged BALB/c mice via improvement of the Th1/Th2 balance toward Th1 dominance [56]. In addition, our previous study demonstrated that supplementation of L9 can protect against the development of Bovine β-Lactoglobulin induced food allergies by modulating the intestinal Th1/Th2 immune response [22]. Taken all together, L9 may attenuate the Th2-related allergic response by balancing an imbalance of Th1/Th2.

Moreover, the present data show that oral administration of higher dose of L9 significantly decreases the level of IL-17 but increases the level of TGF-β in BALF. These results suggest that L9 may attenuate the IL-17 related allergic response by inducing the production of Treg cells and suppressing the differentiation of Th17 cells. Probiotics can stimulate the regulatory functions of dendritic cells (DC) to produce various cytokines, such as IL-10 and TGF-β, which promote the generation of CD4^+^25^+^Foxp3^+^Tregs [57–59]. Meanwhile, Foxp3 can inhibit the RORγt-dependent transcription of IL-17 and the Th17 cell differentiation by directly binding to RORγt [60]. Besides, the induction of CD4^+^25^+^Foxp3^+^Tregs is also a process by which probiotics can inhibit the differentiation of Th1 and Th2 cells, resulting in the suppression of the Th2-type response [61]. Kim et al. proved that oral application of Lcr35 prevented the development of allergic disease by suppressing Th2, Th17, and thymic stromal lymphopoietin (TSLP) responses through a mechanism that may involve CD4^+^25^+^Foxp3^+^Tregs in MLNs [18]. Moreover, according to our previous study, L9 was demonstrated to significantly increase the number of CD4^+^25^+^Foxp3^+^Tregs in the mesenteric lymph node (MLN) and to induce high levels of TGF-β and IL-10 in the serum and lymphocyte supernatants from the MLN [22]. Thus, it can be inferred that oral administration of L9 increases the production of TGF-β in the lung, which may associated with induction of Tregs that contribute to the suppression of IL-17A production and Th2 cell differentiation. However, the details of mechanisms remain to be explored.

In summary, the higher dose of L9 (4×10^9^ CFU/mouse per day) showed significant efficacy to prevent exacerbation of lung inflammatory response in mice. Thus, L9 is a promising candidate for protection from and preventive treatment of allergic disease, especially for individuals suffering from PM_2.5_ exposure enhanced pre-existing asthma.

## Supporting information

S1 Fig Experimental setup of the PM_2.5_ exposure induced mouse model of asthma. (DOCX)

S2 Fig AHR to increasing doses of Mch in mice induced by intranasal administration of PM_2.5_ alone. (DOCX)

S3 Fig Histopathological examination of lung tissue inflammatory cell infiltration in mice induced by intranasal administration of PM_2.5_ alone. Representative photos of H&E-stained lung sections (original magnification 20x).
(DOCX)

## References

[1] Huang RJ, Zhang Y, Bozzetti C, Ho KF, Cao JJ, Han Y, et al High secondary aerosol contribution to particulate pollution during haze events in China. Nature. 2014;514(7521):218–22. 10.1038/nature13774 25231863

[2] Ouyang Y. China wakes up to the crisis of air pollution. Lancet Respir Med. 2013;1(1):12 10.1016/S2213-2600(12)70065-6 24321793

[3] Chen R, Zhao Z, Kan H. Heavy smog and hospital visits in Beijing, China. Am J Respir Crit Care Med. 2013;188(9):1170–1. 10.1164/rccm.201304-0678LE 24180450

[4] Olin JT, Wechsler ME. Asthma: pathogenesis and novel drugs for treatment. BMJ. 2014;349:g5517 10.1136/bmj.g5517 25420994

[5] Martinez FD, Vercelli D. Asthma. The Lancet. 2013;382(9901):1360–72. 10.1016/S0140-6736(13)61536-6 PMC11753400 24041942

[6] Kim HY, DeKruyff RH, Umetsu DT. The many paths to asthma: phenotype shaped by innate and adaptive immunity. Nat Immunol. 2010;11(7):577–84. PubMed Central PMCID: PMC3114595. 10.1038/ni.1892 20562844 PMC3114595

[7] Murphy DM, O'Byrne PM. Recent advances in the pathophysiology of asthma. Chest. 2010;137(6):1417–26. 10.1378/chest.09-1895 20525652

[8] Guarnieri M, Balmes JR. Outdoor air pollution and asthma. The Lancet. 2014;383(9928):1581–92. 10.1016/S0140-6736(14)60617-6 PMC4465283 24792855

[9] Penton PC, Wang X, Amatullah H, Cooper J, Godri K, North ML, et al Spleen tyrosine kinase inhibition attenuates airway hyperresponsiveness and pollution-induced enhanced airway response in a chronic mouse model of asthma. J Allergy Clin Immunol. 2013;131(2):512–20 e1-10. 10.1016/j.jaci.2012.07.039 22981792

[10] Nikasinovic L, Just J, Sahraoui F, Seta N, Grimfeld A, Momas I. Nasal inflammation and personal exposure to fine particles PM2.5 in asthmatic children. J Allergy Clin Immunol. 2006;117(6):1382–8. 10.1016/j.jaci.2006.03.023 16751001

[11] Li N, Harkema JR, Lewandowski RP, Wang M, Bramble LA, Gookin GR, et al Ambient ultrafine particles provide a strong adjuvant effect in the secondary immune response: implication for traffic-related asthma flares. Am J Physiol Lung Cell Mol Physiol. 2010;299(3):L374–83. PubMed Central PMCID: PMC2951067. 10.1152/ajplung.00115.2010 20562226 PMC2951067

[12] Saunders V, Breysse P, Clark J, Sproles A, Davila M, Wills-Karp M. Particulate matter-induced airway hyperresponsiveness is lymphocyte dependent. Environ Health Perspect. 2010;118(5):640–6. PubMed Central PMCID: PMC2866679. 10.1289/ehp.0901461 20061214 PMC2866679

[13] Hastie AT, Moore WC, Meyers DA, Vestal PL, Li H, Peters SP, et al Analyses of asthma severity phenotypes and inflammatory proteins in subjects stratified by sputum granulocytes. J Allergy Clin Immunol. 2010;125(5):1028–36 e13. PubMed Central PMCID: PMC2878277. 10.1016/j.jaci.2010.02.008 20398920 PMC2878277

[14] Al-Ramli W, Prefontaine D, Chouiali F, Martin JG, Olivenstein R, Lemiere C, et al T(H)17-associated cytokines (IL-17A and IL-17F) in severe asthma. J Allergy Clin Immunol. 2009;123(5):1185–7. 10.1016/j.jaci.2009.02.024 19361847

[15] Lim LH, Li HY, Huang CH, Lee BW, Lee YK, Chua KY. The effects of heat-killed wild-type Lactobacillus casei Shirota on allergic immune responses in an allergy mouse model. Int Arch Allergy Immunol. 2009;148(4):297–304. 10.1159/000170383 19001789

[16] Forsythe P. Probiotics and lung diseases. Chest. 2011;139(4):901–8. 10.1378/chest.10-1861 21467057

[17] Julia V, Macia L, Dombrowicz D. The impact of diet on asthma and allergic diseases. Nat Rev Immunol. 2015;15(5):308–22. 10.1038/nri3830 25907459

[18] Kim HJ, Kim YJ, Lee SH, Yu J, Jeong SK, Hong SJ. Effects of Lactobacillus rhamnosus on allergic march model by suppressing Th2, Th17, and TSLP responses via CD4(+)CD25(+)Foxp3(+) Tregs. Clin Immunol. 2014;153(1):178–86. 10.1016/j.clim.2014.04.008 24769377

[19] Fujiwara D, Inoue S, Wakabayashi H, Fujii T. The anti-allergic effects of lactic acid bacteria are strain dependent and mediated by effects on both Th1/Th2 cytokine expression and balance. Int Arch Allergy Immunol. 2004;135(3):205–15. 10.1159/000081305 15467373

[20] Jang SO, Kim HJ, Kim YJ, Kang MJ, Kwon JW, Seo JH, et al Asthma Prevention by Lactobacillus Rhamnosus in a Mouse Model is Associated With CD4(+)CD25(+)Foxp3(+) T Cells. Allergy Asthma Immunol Res. 2012;4(3):150–6. PubMed Central PMCID: PMC3328732. 10.4168/aair.2012.4.3.150 22548208 PMC3328732

[21] Jan RL, Yeh KC, Hsieh MH, Lin YL, Kao HF, Li PH, et al Lactobacillus gasseri suppresses Th17 pro-inflammatory response and attenuates allergen-induced airway inflammation in a mouse model of allergic asthma. Br J Nutr. 2012;108(1):130–9. 10.1017/S0007114511005265 21996276

[22] Luo X, Hao Y, Jiang L, Zhang H, Ren F, Yang J. Induction of Regulatory Dendritic Cells by Lactobacillus paracasei L9 Prevents Allergic Sensitization to Bovine β-Lactoglobulin in Mice. Journal of Microbiology and Biotechnology. 2015;25(10):1687–96. 10.4014/jmb.1503.03022 26095382

[23] Mazzoli-Rocha F, Magalhaes CB, Malm O, Saldiva PH, Zin WA, Faffe DS. Comparative respiratory toxicity of particles produced by traffic and sugar cane burning. Environ Res. 2008;108(1):35–41. 10.1016/j.envres.2008.05.004 18606401

[24] Yao XJ, Huang KW, Li Y, Zhang Q, Wang JJ, Wang W, et al Direct comparison of the dynamics of IL-25- and ‘allergen’-induced airways inflammation, remodelling and hypersensitivity in a murine asthma model. Clin Exp Allergy. 2014;44(5):765–77. 10.1111/cea.12298 24575868

[25] Kim H, Kwack K, Kim D-Y, Ji GE. Oral probiotic bacterial administration suppressed allergic responses in an ovalbumin-induced allergy mouse model. FEMS Immunology & Medical Microbiology. 2005;45(2):259–67. 15963706 10.1016/j.femsim.2005.05.005

[26] Jang S-O, Kim H-J, Kim Y-J, Kang M-J, Kwon J-W, Seo J-H, et al Asthma prevention by Lactobacillus rhamnosus in a mouse model is associated with CD4+ CD25+ Foxp3+ T cells. Allergy, asthma & immunology research. 2012;4(3):150–6. 10.4168/aair.2012.4.3.150 PMC3328732 22548208

[27] Shibamori M, Ogino K, Kambayashi Y, Ishiyama H. Intranasal mite allergen induces allergic asthma-like responses in NC/Nga mice. Life sciences. 2006;78(9):987–94. 10.1016/j.lfs.2005.06.020 16229861

[28] Molet S, Hamid Q, Davoine F, Nutku E, Taha R, Page N, et al IL-17 is increased in asthmatic airways and induces human bronchial fibroblasts to produce cytokines. J Allergy Clin Immun. 2001;108(3):430–8. 10.1067/mai.2001.117929 11544464

[29] Laan M, Cui ZH, Hoshino H, Lotvall J, Sjostrand M, Gruenert DC, et al Neutrophil recruitment by human IL-17 via C-X-C chemokine release in the airways. J Immunol. 1999;162(4):2347–52. 9973514

[30] Triggianese P, Conigliaro P, Chimenti MS, Biancone L, Monteleone G, Perricone R, et al Evidence of IL-17 producing innate lymphoid cells in peripheral blood from patients with enteropathic spondyloarthritis. Clinical and experimental rheumatology. 2016;34(6):1085–93. 27782868

[31] Ferretti S, Bonneau O, Dubois GR, Jones CE, Trifilieff A. IL-17, produced by lymphocytes and neutrophils, is necessary for lipopolysaccharide-induced airway neutrophilia: IL-15 as a possible trigger. J Immunol. 2003;170(4):2106–12. 12574382 10.4049/jimmunol.170.4.2106

[32] Weaver CT, Hatton RD, Mangan PR, Harrington LE. IL-17 family cytokines and the expanding diversity of effector T cell lineages. Annual review of immunology. 2007;25:821–52. 10.1146/annurev.immunol.25.022106.141557 17201677

[33] Weaver CT, Harrington LE, Mangan PR, Gavrieli M, Murphy KM. Th17: an effector CD4 T cell lineage with regulatory T cell ties. Immunity. 2006;24(6):677–88. 10.1016/j.immuni.2006.06.002 16782025

[34] Schmidt-Weber CB, Akdis M, Akdis CA. TH17 cells in the big picture of immunology. J Allergy Clin Immunol. 2007;120(2):247–54. 10.1016/j.jaci.2007.06.039 17666214

[35] Brandt EB, Kovacic MB, Lee GB, Gibson AM, Acciani TH, Le Cras TD, et al Diesel exhaust particle induction of IL-17A contributes to severe asthma. J Allergy Clin Immunol. 2013;132(5):1194–204 e2. PubMed Central PMCID: PMC3845500. 10.1016/j.jaci.2013.06.048 24060272 PMC3845500

[36] Pappu R, Rutz S, Ouyang W. Regulation of epithelial immunity by IL-17 family cytokines. Trends Immunol. 2012;33(7):343–9. 10.1016/j.it.2012.02.008 22476048

[37] Wakashin H, Hirose K, Maezawa Y, Kagami S, Suto A, Watanabe N, et al IL-23 and Th17 cells enhance Th2-cell-mediated eosinophilic airway inflammation in mice. Am J Respir Crit Care Med. 2008;178(10):1023–32. 10.1164/rccm.200801-086OC 18787221

[38] Kudo M, Melton AC, Chen C, Engler MB, Huang KE, Ren X, et al IL-17A produced by alphabeta T cells drives airway hyper-responsiveness in mice and enhances mouse and human airway smooth muscle contraction. Nat Med. 2012;18(4):547–54. PubMed Central PMCID: PMC3321096. 10.1038/nm.2684 22388091 PMC3321096

[39] Gavett SH, Madison SL, Dreher KL, Winsett DW, McGee JK, Costa DL. Metal and sulfate composition of residual oil fly ash determines airway hyperreactivity and lung injury in rats. Environ Res. 1997;72(2):162–72. 10.1006/enrs.1997.3732 9177658

[40] Lambert AL, Selgrade MJ, Dong W, Winsett DW, Gilmour MI. Enhanced allergic sensitization by residual oil fly ash particles is mediated by soluble metal constituents. Am J Respir Crit Care Med. 1999;159(3):A26–A. 10.1006/taap.2000.8932 10814556

[41] Carter JD, Ghio AJ, Samet JM, Devlin RB. Cytokine production by human airway epithelial cells after exposure to an air pollution particle is metal-dependent. Toxicol Appl Pharmacol. 1997;146(2):180–8. 10.1006/taap.1997.8254 9344885

[42] Heinrich J, Hoelscher B, Wjst M, Ritz B, Cyrys J, Wichmann H. Respiratory diseases and allergies in two polluted areas in East Germany. Environ Health Perspect. 1999;107(1):53–62. PubMed Central PMCID: PMC1566314. 9872717 10.1289/ehp.9910753 PMC1566314

[43] Wagner JG, Morishita M, Keeler GJ, Harkema JR. Divergent effects of urban particulate air pollution on allergic airway responses in experimental asthma: a comparison of field exposure studies. Environ Health. 2012;11:45 PubMed Central PMCID: PMC3487754. 10.1186/1476-069X-11-45 22768850 PMC3487754

[44] Gavett SH, Haykal-Coates N, Copeland LB, Heinrich J, Gilmour MI. Metal Composition of Ambient PM2.5 Influences Severity of Allergic Airways Disease in Mice. Environ Health Perspect. 2003;111(12):1471–7. 12948886 10.1289/ehp.6300 PMC1241649

[45] Ogino K, Zhang R, Takahashi H, Takemoto K, Kubo M, Murakami I, et al Allergic airway inflammation by nasal inoculation of particulate matter (PM2.5) in NC/Nga mice. PloS one. 2014;9(3):e92710 PubMed Central PMCID: PMC3966822. 10.1371/journal.pone.0092710 24671176 PMC3966822

[46] Kimura A, Naka T, Nohara K, Fujii-Kuriyama Y, Kishimoto T. Aryl hydrocarbon receptor regulates Stat1 activation and participates in the development of Th17 cells. Proc Natl Acad Sci U S A. 2008;105(28):9721–6. PubMed Central PMCID: PMC2474493. 10.1073/pnas.0804231105 18607004 PMC2474493

[47] Veldhoen M, Hirota K, Westendorf AM, Buer J, Dumoutier L, Renauld JC, et al The aryl hydrocarbon receptor links TH17-cell-mediated autoimmunity to environmental toxins. Nature. 2008;453(7191):106–9. 10.1038/nature06881 18362914

[48] van Voorhis M, Knopp S, Julliard W, Fechner JH, Zhang X, Schauer JJ, et al Exposure to atmospheric particulate matter enhances Th17 polarization through the aryl hydrocarbon receptor. PloS one. 2013;8(12):e82545 PubMed Central PMCID: PMC3859609. 10.1371/journal.pone.0082545 24349309 PMC3859609

[49] Laake KO, Bjorneklett A, Aamodt G, Aabakken L, Jacobsen M, Bakka A, et al Outcome of four weeks' intervention with probiotics on symptoms and endoscopic appearance after surgical reconstruction with a J-configurated ileal-pouch-anal-anastomosis in ulcerative colitis. Scandinavian journal of gastroenterology. 2005;40(1):43–51. 15841713 10.1080/00365520410009339

[50] Rosenfeldt V, Benfeldt E, Nielsen SD, Michaelsen KF, Jeppesen DL, Valerius NH, et al Effect of probiotic Lactobacillus strains in children with atopic dermatitis. J Allergy Clin Immunol. 2003;111(2):389–95. 12589361 10.1067/mai.2003.389

[51] Pessi T, Sutas Y, Hurme M, Isolauri E. Interleukin-10 generation in atopic children following oral Lactobacillus rhamnosus GG. Clin Exp Allergy. 2000;30(12):1804–8. 11122221 10.1046/j.1365-2222.2000.00948.x

[52] Aldinucci C, Bellussi L, Monciatti G, Passali GC, Salerni L, Passali D, et al Effects of dietary yoghurt on immunological and clinical parameters of rhinopathic patients. European journal of clinical nutrition. 2002;56(12):1155–61. 10.1038/sj.ejcn.1601465 12494299

[53] Hong HJ, Kim E, Cho D, Kim TS. Differential suppression of heat-killed lactobacilli isolated from kimchi, a Korean traditional food, on airway hyper-responsiveness in mice. Journal of clinical immunology. 2010;30(3):449–58. 10.1007/s10875-010-9375-8 20204477

[54] Feleszko W, Jaworska J, Rha RD, Steinhausen S, Avagyan A, Jaudszus A, et al Probiotic-induced suppression of allergic sensitization and airway inflammation is associated with an increase of T regulatory-dependent mechanisms in a murine model of asthma. Clin Exp Allergy. 2007;37(4):498–505. 10.1111/j.1365-2222.2006.02629.x 17430345

[55] Hougee S, Vriesema AJ, Wijering SC, Knippels LM, Folkerts G, Nijkamp FP, et al Oral treatment with probiotics reduces allergic symptoms in ovalbumin-sensitized mice: a bacterial strain comparative study. Int Arch Allergy Immunol. 2010;151(2):107–17. 10.1159/000236000 19752564

[56] Liu YW, Liao TW, Chen YH, Chiang YC, Tsai YC. Oral administration of heat-inactivated Lactobacillus plantarum K37 modulated airway hyperresponsiveness in ovalbumin-sensitized BALB/c mice. PloS one. 2014;9(6):e100105 PubMed Central PMCID: PMC4061068. 10.1371/journal.pone.0100105 24936861 PMC4061068

[57] Kwon HK, Lee CG, So JS, Chae CS, Hwang JS, Sahoo A, et al Generation of regulatory dendritic cells and CD4(+)Foxp3(+) T cells by probiotics administration suppresses immune disorders. Proc Natl Acad Sci U S A. 2010;107(5):2159–64. 10.1073/pnas.0904055107 20080669 PMC2836639

[58] Puccetti P, Grohmann U. IDO and regulatory T cells: a role for reverse signalling and non-canonical NF-kappa B activation. Nature Reviews Immunology. 2007;7(10):817–23. 10.1038/nri2163 17767193

[59] Hart AL, Lammers K, Brigidi P, Vitali B, Rizzello F, Gionchetti P, et al Modulation of human dendritic cell phenotype and function by probiotic bacteria. Gut. 2004;53(11):1602–9. 10.1136/gut.2003.037325 15479680 PMC1774301

[60] Zhou L, Lopes JE, Chong MMW, Ivanov II, Min R, Victora GD, et al TGF-beta-induced Foxp3 inhibits T(H)17 cell differentiation by antagonizing ROR gamma t function. Nature. 2008;453(7192):236–U14. 10.1038/nature06878 18368049 PMC2597437

[61] Belkaid Y. The role of CD4(+)CD25(+) regulatory T cells in Leishmania infection. Expert Opin Biol Ther. 2003;3(6):875–85. 10.1517/14712598.3.6.875 12943446

