# Supplementary figures and images for: Oral administration of Lactobacillus paracasei L9 attenuates PM2.5-induced enhancement of airway hyperresponsiveness and allergic airway response in murine model of asthma
Source: PLoS One. 2017 Feb 15;12(2):e0171721. doi: 10.1371/journal.pone.0171721 (PMC5310903; doi:10.1371/journal.pone.0171721)

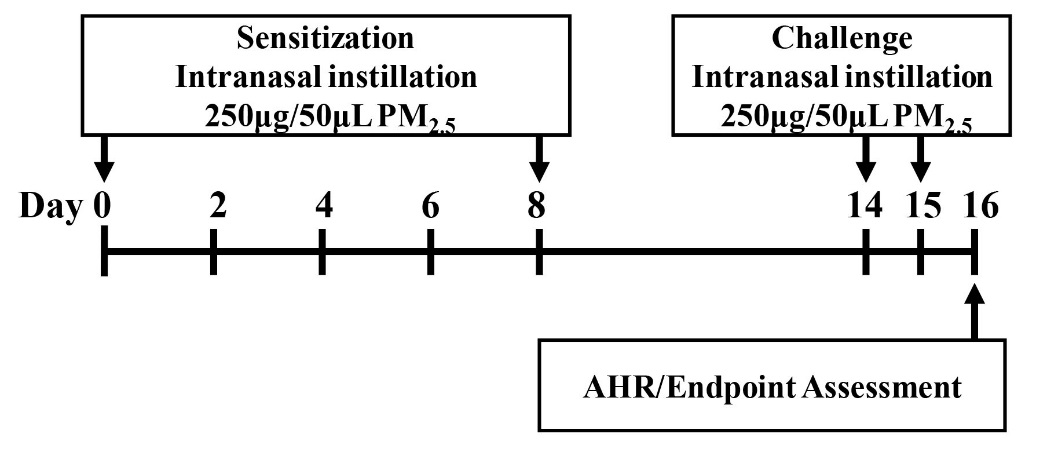


**S1 Fig. Experimental setup of the PM2.5 exposure induced mouse model of asthma.**

Supplement: S1 Fig — (DOCX) [file pone.0171721.s001.docx]

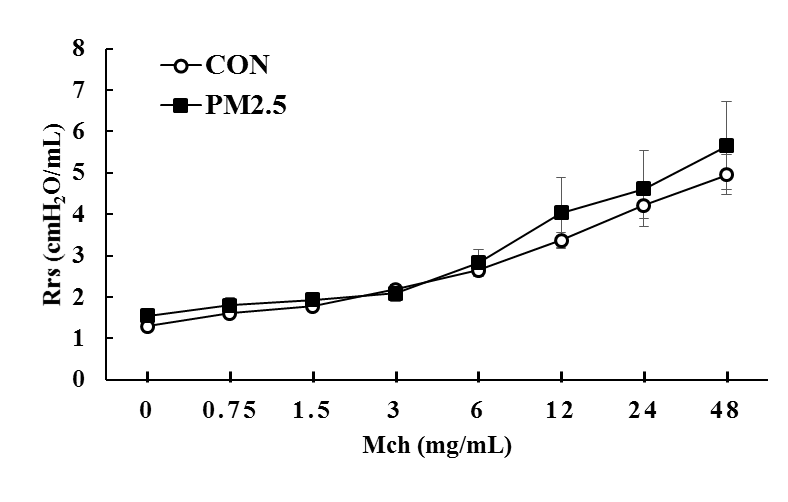


**S2 Fig. AHR to increasing doses of Mch in mice induced by intranasal administration of PM2.5 alone.**

Supplement: S2 Fig — (DOCX) [file pone.0171721.s002.docx]
