# Supplementary material for: Oral administration of Lactobacillus paracasei L9 attenuates PM2.5-induced enhancement of airway hyperresponsiveness and allergic airway response in murine model of asthma
Source: PLoS One. 2017 Feb 15;12(2):e0171721. doi: 10.1371/journal.pone.0171721 (PMC5310903; doi:10.1371/journal.pone.0171721)
Supplement: S3 Fig — Representative photos of H&E-stained lung sections (original magnification 20x). (DOCX) [file pone.0171721.s003.docx]

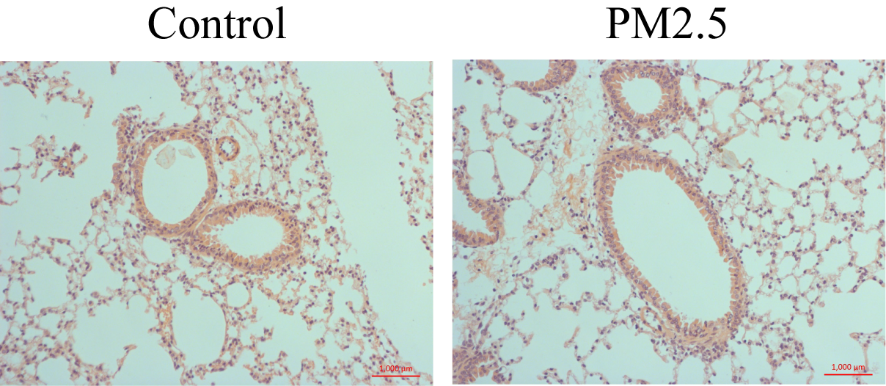


**S3 Fig. Histopathological examination of lung tissue inflammatory cell infiltration in mice induced by intranasal administration of PM_2.5_ alone.** Representative photos of H&E-stained lung sections (original magnification 20x).
